# Relevance of Serum Levels of Interleukin-6 and Syndecan-1 in Patients with Hepatocellular Carcinoma

**DOI:** 10.3797/scipharm.1110-07

**Published:** 2011-12-23

**Authors:** Heba A. Metwaly, Mohammed M. H. Al-Gayyar, Shahira Eletreby, Mohamed A. Ebrahim, Mamdouh M. El-Shishtawy

**Affiliations:** 1 Department of Biochemistry, Faculty of Pharmacy, Mansoura University, Mansoura, 35516, Egypt; 2 Department of Internal Medicine, Faculty of Medicine, Mansoura University, Mansoura, 35516, Egypt; 3 Oncology Center, Mansoura University, Mansoura, 35516, Egypt

**Keywords:** Syndecan-1, IL-6, HCC, Liver cirrhosis, α-Fetoprotein

## Abstract

Syndecan-1 is a trans-membrane heparan sulfate proteoglycan that localizes in epithelial cells and has been shown to be present in normal hepatocytes. It is thought to be involved in processes such as cell growth, differentiation and adhesion. However, the clinical data regarding syndecan-1 in patients with hepatocellular carcinoma (HCC) are scarce and controversial. Therefore, we need to evaluate the effects of HCC on the serum levels of syndecan-1. Thus, 40 patients with HCC and 31 patients with liver cirrhosis were physically examined. Blood samples were taken for measurements of routine markers (sGPT, sGOT, bilirubin, albumin, and α-fetoprotein), as well as serum levels of interleukin (IL)-6 and syndecan-1. Patients with liver cirrhosis showed significant increase in serum IL-6 as compared with HCC patients and the control subjects. Serum level of syndecan-1 was significantly increased in HCC patients as compared with the cirrhotic and control groups. In addition, significant positive correlations between syndecan-1 and serum levels of ALT, AST in HCC patients were found. Moreover, syndecan-1 increased significantly with increasing stage of Barcelona-Clinic Liver Cancer Group diagnostic and treatment strategy. In conclusion, the development of HCC is accompanied by a significant elevation in serum syndecan-1 levels. The increase in serum syndecan-1 may be linked with progression of HCC.

## Introduction

Hepatocellular carcinoma (HCC) has become the third most common malignancy worldwide with very poor prognosis, rendering it the fourth highest cause of cancer-related deaths. In Africa, liver cancer has been ranked as the fourth common cancer, and most of liver cancers are HCC [1]. In addition, Egypt has the highest prevalence of HCV worldwide and has rising rates of HCC. HCC comprises nearly 6% of all incident cancer cases worldwide. Moreover, HCC is the second most frequent cause of cancer incidence and mortality among men in Egypt. Hospital-based studies from Egypt have reported an increase in the relative frequency of all liver-related cancers in Egypt (>95% as HCC), from 4.0% in 1993 to 7.3% in 2003 [2].

It is very important to detect HCC and the recurrence at its earlier period. By reasons of convenience, inexpensiveness, and the satisfactory accuracy, serum tumor markers have been used as an effective method for detecting malignant tumors for a long time, and they could be valuable supplementary to ultrasonography and computer tomography in the diagnosis of HCC. Using the appropriate single or combination of tumor markers may improve the effectiveness in screening HCC patients [3]. Currently, standard surveillance includes a combination of 6 monthly ultrasound scans (USS) and serum α-fetoprotein measurements, but this strategy dose not reliably detect the disease in its early stages [4]. Therefore, it is very clear that there is an imperious need for newer markers with greater accuracy in diagnosis of early HCC.

Interleukin (IL)-6 is a glycoprotein of 21–28 kDa molecular weight and consists of 184 amino acids [5]. IL-6 is a pleiotropic cytokine that plays a central role in hematopoiesis, as well as in the differentiation and growth of a number of cells of different histologic origin, e.g. endothelial cells, keratinocytes, neuronal cells, osteoclasts, and osteoblasts. Moreover, IL-6 induces the hepatic acute phase response by modulating the transcription of several liver-specific genes during inflammation [6].

Syndecan-1, a trans-membrane heparan sulfate proteoglycan, localizes in epithelial cells and has been shown to be present in normal hepatocytes. It interacts with growth factors, matrix components and other extracellular proteins and is thought to be involved in processes such as cell growth, differentiation and adhesion [7]. Syndecan-1 plays roles in early development and wound healing [8]. The expression of syndecan-1 appears generally down-regulated in human carcinomas and in experimental cancer models, whereas transfectional expression of syndecan-1 in cultured cancer cells has been shown to inhibit their growth and other aspects of malignant behavior [7].

Altered levels of the pro-inflammatory cytokines, IL-6, have been associated with morbidity and disease activity in an astonishing variety of cancers. In light of these observations, we tried to explore the prognostic role of IL-6 in patients with HCC that might be correlated with clinical efficacy by comparing clinical outcome. In addition, the role of syndecan-1 in regulating inflammation in a variety of disease models including cancer has been previously reported [9]. Therefore, the prognostic role syndecan-1 in patients with HCC will be also evaluated.

## Experimental

### Patients

From August to December 2010, 40 patients with HCC (9 ♀ and 31 ♂; aged 45–80 years with a mean±SE of 57.6±1.39) were selected from the Oncology Unit, Mansoura University, Mansoura, Egypt. Since all HCC patients in this study have cirrhosis as underlying liver disorder and in order to nullify the effect of cirrhosis on the level of studied parameters, a group of 31 cirrhotic patients (5 ♀ and 26 ♂; aged 38–59 years with a mean±SE of 53.7±1.76) without any evidence of HCC was used and selected from the in-patient Clinics of Specialized Medical Hospital, Mansoura University, Mansoura, Egypt. The study was approved by the local institutional ethical committee and patients’ consents were obtained according to the regulations of the Egyptian Ministry of Health. All cases involved in this study were clinically, radiologically and pathologically examined in the Oncology Unit and the Specialized Medical Hospital, Mansoura University. HCC was diagnosed by abdominal ultrasonography and serum AFP, with or without triphasic computed tomography scan and/or liver histopathology. Severity of liver disease was assessed by Child-Pugh classification [10]. The stage and management were defined according to the Barcelona-Clinic Liver Cancer Group diagnostic and treatment strategy (BCLC) [11].

Patients with other types of malignancy, advanced organ failure, active infection and advanced medical co-morbidity were excluded from the study.

### Control group

The control group consisted of 15 healthy individuals (4 ♀ and 11 ♂; aged 30–64 years with a mean±SE of 50.7±2.7) with no apparent evidence of active disease or medical disorders.

### Blood sampling

5 ml of fasting blood was collected by vein puncture from each patient and control subject and subsequently divided into two portions. The first portion (1 ml) was collected in tubes containing ethylene diamine tetra acetic acid (EDTA) and was used for blood picture investigation within 5 h. The second portion (4 ml) was left to clot for 20–30 min at room temperature and followed by centrifugation at 1500 r.p.m for 10 min. The serum was then transferred to a polypropylene tube and if the analysis was not performed immediately, the samples were frozen and maintained at −80°C until use.

### Analysis of biochemical parameters

Serum ALT, AST, alkaline phosphatase, γ-Glutamyl transferase activities as well as albumin and total bilirubin concentrations were measured using standard methodologies. Hemoglobin concentration was determined by a colorimetric method. Red blood corpuscles, white blood cells and platelets were counted by hemocytometer apparatus.

Serum α-fetoprotein, Interleukin-6 and syndecan-1 concentrations were measured using commercially available ELIZA kits from (Ameritek USA company, Orgenium Laboratories Business Unit and Cell Science Company, respectively).

### Statistical analysis

For descriptive statistics the frequency and percentage were calculated for qualitative variables, the mean values ± standard deviation (SD), standard error (SE), and range were used for quantitative variables. For comparison between two groups student t-test was used. For correlation, GraphPad Instat version 3.06 was used. Statistical computations were done using Excel 2007 and statistical significance was predefined as P ≤ 0.05.

## Results and Discussion

In the present study, we measured serum IL-6 and syndecan-1 levels in patients with HCC and healthy controls and evaluated a possible prognostic value of these markers. In most solid malignancies, tumor stage at presentation determines prognosis and plan of management. However, most patients with HCC have two diseases, liver cirrhosis and HCC, and complex interactions between the two have major implications for prognosis and treatment choice. Our study included 40 patients with HCC, and all of them are cirrhotics. Therefore, 31 patients with liver cirrhosis were included in the study. All the patients were subjected to clinical evaluation and history taking as well as measurements of serum levels of liver function. Patients’ characteristics were summarized in table 1. The liver functions of patients were summarized in table 2.

We found a significant increase in serum concentration of α-fetoprotein in HCC patients as compared to patients with liver cirrhosis and the control group. Similar results were obtained by many studies [12–15]. Some reports have indicated that α-fetoprotein has limited utility of differentiating HCC from benign hepatic disorders for its high false-positive and false-negative rates, and patients with acute exacerbation of viral hepatitis but no HCC may also have markedly increased α-fetoprotein levels [3].

IL-6 is a multifunctional cytokine with both differentiation and growth-promoting effects for target cells. IL-6 regulates the synthesis of a broad spectrum of acute-phase proteins in the liver. Up-regulated expression of the IL-6 gene appears to be involved in pathological conditions, and it has also been hypothesized that activation of IL-6 gene might trigger initial events leading to oncogenic transformation [16]. IL-6 was postulated to correlate with the stage of liver cirrhosis [6, 17]. Therefore, we aimed to measure the serum concentration IL-6 in patients with liver cirrhosis and HCC to evaluate its activity as tumor marker for liver malignancies.

As shown in table 3, we found a significant increase in serum IL-6 in HCC patients as compared with control subjects similar to the results of Ataseven [18]. Furthermore, we found a significant decrease in serum IL-6 concentration in HCC patients as compared to patients with liver cirrhosis. This result coincided with those recorded by Lee [16]. However, Soresi [17] and Porta [6] found that there was a significant increase in serum IL-6 concentration of HCC patients as compared to cirrhotic patients. However, we found that HCC patients showed increased serum IL-6 concentration with increased BCLC score (Tables 4, 6). Moreover, serum IL-6 level was positively correlated with serum α-fetoprotein (Table 5). In agreement, Soresi [17] suggested that HCC cells, especially in advanced stages of the disease, may produce and secrete IL-6 and its receptor to stimulate their growth by an autocrine/paracrine mechanism. It has been previously shown that serum IL-6 was increased in patients with liver diseases like fatty liver even before any increase in serum level of liver enzymes [19].

Syndecan-1 is the main heparan sulphate present on the surface of plasma cells. Syndecan-1 binds to extracellular matrix proteins and to growth factors that have a HS-binding domain. Syndecan-1 mediates hepatocyte growth factor (HGF) binding and promotes its signaling [20]. Via its heparan sulphate chains, Syndecan-1 binds to a variety of growth and angiogenic factors and acts as a classical co-receptor for growth factor receptors, thus promoting cell proliferation. Moreover, Syndecan-1 interacts with ligands in the extracellular matrix and on cell surfaces, functioning as a cell adhesion molecule. It has been previously shown that Syndecan-1 is a modulator of proteolytic activities and chemokine functions in vivo, which regulates leucocyte recruitment and tissue remodeling during inflammation and wound repair [21].

Syndecan-1 plays an important role in the growth, survival, vasculogenesis, and metastasis of various cancers, including myelomas and breast, bladder, ovary, prostate and colon cancers. Syndecan-1 mRNA levels are up-regulated in pancreatic cancer in association with accelerated tumor growth [22]. As a result, we conducted the following research to measure the effect of liver cirrhosis or HCC on the serum level of syndecan-1.

We found a significant increase in serum syndecan-1 concentration in cirrhotic and HCC patients in comparison with liver cirrhosis patients or the control subjects (Table 3). In addition, significant positive correlations between syndecan-1 and serum activities of ALT and AST in HCC patients were found (Table 5). Moreover, syndecan-1 increased significantly with increasing stage of BCLC (Tables 4, 6). To our knowledge, this is the first study which measured serum level of syndecan-1 in patients with HCC.

It has been reported previously that proteolytic conversion of syndecan-1 from a membrane-bound into a soluble molecule marks a switch from a proliferative to an invasive tumor [23]. Soluble (shed) syndecan-1 can actively promote myeloma tumor growth, angiogenesis and metastasis. The finding that heparanase promotes syndecan-1 synthesis and shedding suggests that these two molecules act synergistically to fuel and accelerate tumor progression [24]. The proteolytic cleavage of syndecan-1 is mediated by a variety of proteases of the matrix metalloproteinase (MMP) family [23]. MMP7 is frequently over expressed by fibroblast growth factor (FGF)-2 [25]. Off note, FGF-2 concentration was elevated prior to the emergence of HCC [26]. Therefore, FGF-2/MMP7 axis may be implicated in up-regulation of serum levels of syndecan-1 in HCC, which may reflect the aggressive character of HCC. However, further studies should be performed.

## Conclusions

This study demonstrated that serum syndecan-1 levels are significantly elevated in patients with HCC, and this elevation correlates with advanced stage (BCLC) of the tumor. Therefore, we can conclude that:

1. Serum syndecan-1 is a promising diagnostic marker for HCC that may aid in screening and detection of HCC.
2. Serum syndecan-1 is a potential prognostic marker for HCC, linked with advanced stage of the disease.
3. These findings should be validated in further prospective and longitudinal studies recruiting a large number of patients.

## Figures and Tables

**Tab. 1:** Patients characteristics

		HCC (n=40)	Cirrhosis (n=31)
Sex	Male	31 (77.5%)	26 (83.9%)
	Female	9 (22.5%)	5 (16.1%)

Diabetes	No	27 (67.5%)	21 (67.7%)
	Yes	13 (32.5%)	10 (32.3%)

Hypertension	No	29 (72.5%)	22 (71%)
	Yes	11 (27.5%)	9 (29%)

Ascitis	No	18 (45%)	15 (48.4%)
	Yes	22 (55%)	16 (52.8%)

Etiology of Liver Disease	HCV	25 (62.5%)	23 (74.2%)
	HBV	8 (20%)	2 (6.5%)
	Both HCV & HBV(+)	4 (10%)	2 (6.5%)
	Other	3 (7.5%)	4 (12.9%)

Child	A	14 (35%)	11 (35.4%)
	B	20 (50%)	14 (45.2%)
	C	6 (15%)	6 (19.4%)

BCLC	A	12 (30%)	–
	B	14 (35%)	
	C	8 (20%)	
	D	6 (15%)	

^n^ number of patients; BCLC…Barcelona-Clinic Liver Cancer Group diagnostic and treatment strategy.

**Tab. 2:** Liver function tests of patients with cirrhosis and HCC as compared with the control subjects (mean±SE).

	Control group (n=15)	Cirrhotic group (n=31)	HCC group (n=40)
ALT (U/ml)	8.57±0.65	34.93±3.12*	67.47±4.51*#
AST (U/ml)	13.79±1.26	62.95±5.45*	78.13±3.99*#
Total bilirubin (mg/dl)	0.69±0.01	3.31±0.34*	1.26±0.09*#
Albumin (g/dl)	4.33±0.15	2.92±0.13*	3.09±0.1*
ALP (U/l)	67.55±3.56	82.99±4.99*	120.79±7.87*#
γGT (U/l)	20.53±1.88	48.37±4.6*	99.54±9.96*#

^n^ number of patients;

* significant difference as compared with the control group at p<0.05;

# significant difference as compared with the cirrhotic group at p<0.05.

**Tab. 3:** Serum concentration of α-fetoprotein, IL-6 and syndecan-1 in cirrhotic and HCC patients as compared with the control group (mean±SE).

	Control group (n=15)	Cirrhotic group (n=31)	HCC group (n=40)
α-fetoprotein (ng/ml)	16.06±1.59	118.5±9.65*	450.51±40.93*#
IL-6 (pg/ml)	69.75±0.83	132.46±11.44*	86.97±6.17*#
Syndecan-1 (ng/ml)	31.52±15.3	83.23±10.13*	128.64±16.45*#

^n^ number of patients;

* significant difference as compared with the control group at p<0.05;

# significant difference as compared with the cirrhotic group at p<0.05.

**Tab. 4:** Serum concentration of IL-6 and syndecan-1 in HCC patients in relation to BCLC staging (mean±SE).

	A (n=12)	B (n=14)	C & D (n=14)
IL-6 (pg/ml)	75.39±2.05	82.18±3.08*	96.06±5.37#
Syndecan-1 (ng/ml)	39.4±3.58	113.24±7.57*	264.69±37.37#

^n^ number of patients;

* significant difference as compared with BCLC A group at p<0.05;

# significant difference as compared with BCLC B group at p<0.05.

**Tab. 5:** Correlation between serum syndecan-1 and IL-6 with the measured parameters in cirrhotic and HCC patients.

	Syndecan-1		IL-6	
	
Parameters	Cirrhotic patients	HCC patients	Cirrhotic patients	HCC patients
ALT	−0.04	0.61*	−0.05	0.08
AST	0.52*	0.55*	0.24*	0.69*
Total bilirubin	0.39*	0.09	0.36*	−0.21
Albumin	0.08	−0.23*	−0.41*	0.20
Alkaline phosphatase	0.23	−0.08	0.06	−0.10
γ-glutamyl transferase	0.07	−0.09	−0.1	−0.13
α-fetoprotein	0.38*	0.28*	0.4*	0.4*
IL-6	0.49*	0.04		

* significant difference at p<0.05.

**Tab. 6:** Multivariate analysis of serum concentration of α-fetoprotein, IL-6 and syndecan-1 in HCC patients in relation to BCLC staging.

Variable	B	Odd’s Ratio	95% CI
IL-6 (pg/ml)	1.8	1.05*	1.01–2.9
Syndecan-1 (ng/ml)	1.2	1.1*	1.07–3.2
α-fetoprotein (ng/ml)	4.1	1.7	0.2–8.3

* significant at p<0.05.
